# Assessing the determinants of Ebola virus disease transmission in Baka Community of the Tropical Rainforest of Cameroon

**DOI:** 10.1186/s12879-021-06011-z

**Published:** 2021-04-07

**Authors:** Frankline Sevidzem Wirsiy, Alphonse Um Boock, Jane-Francis Tatah Kihla Akoachere

**Affiliations:** 1 Department of Microbiology and Parasitology, Faculty of Science, University of Buea, Buea, Cameroon; 2 FAIRMED, Yaounde, Cameroon

**Keywords:** Ebola virus disease, Knowledge, Practices, Determinants, Misconceptions, Transmission, Cameroon

## Abstract

**Background:**

Ebola virus disease (EVD) is a severe, often fatal illness in humans and nonhuman primates caused by the Ebola virus. The recently approved rVSV-EBOV vaccine is not available in many high-risk countries hence prevention is paramount. The design of effective prevention interventions requires an understanding of the factors that expose communities at risk. It was based on this that we investigated the Baka community of Abong-Mbang Health District in tropical rain forest of Cameroon.

**Methods:**

A cross-sectional study was conducted with participants randomly selected from 13 villages in Abong-Mbang by multi-stage cluster sampling. A questionnaire was administered to them to collect demographic information, data on knowledge of EVD, their feeding and health-seeking behaviour. Data was analyzed using the chi-square test. Knowledge of EVD was assessed using an 8 item Morisky Scale. An adapted Threat Capability Basic Risk Assessment Guide was used to determine their risk of exposure to infection.

**Results:**

A total of 510 participants, most of whom were hunters (31.4%), farmers (29.8%), and had primary education (62.7%), were included in this study. Although 83.3% participants had heard of EVD, most (71%) did not know its cause. Their source of information was mainly informal discussions in the community (49%). Misconceptions were identified with regards to the cause and mode of transmission. Only 43.1% accepted EVD could be transmitted from human-to-human. Generally, participants’ knowledge of EVD was poor. Demographic factors such as level of education, occupation and ethnic group significantly affected knowledge of EVD. The majority of participants were at a very high risk of exposure to infection as they consumed various forms of bush meat and were involved in other risky practices such as scarification and touching of corpses. Although over half of participants seek medical care, most of them preferred traditional medicine. Socio-cultural and service-related factors were deterrent factors to medical care.

**Conclusion:**

Participants generally had poor knowledge of EVD and were at high risk of infection. We recommend rigorous sensitization campaigns in the study area to educate the population on EVD and clarify the misconceptions identified. EVD surveillance is recommended particularly as outbreaks have often been reported in the Congo Basin.

**Supplementary Information:**

The online version contains supplementary material available at 10.1186/s12879-021-06011-z.

## Background

Ebola virus disease (EVD) is a fatal illness affecting humans and nonhuman primates caused by tEbola virus, a member of the family *Filoviridae*. The disease in humans begins with flu-like symptoms. Haemorrhagic symptoms usually appear late resulting in delayed diagnosis [1]. There is increasing evidence of asymptomatic infections [2–4]. Outbreaks in humans have been caused by four of the six species of Ebola virus: Zaire, Bundibugyo, Taї Forest and Sudan [5]. Ebola virus was discovered during simultaneous outbreaks of febrile illness with shock and hemorrhage in Sudan and Democratic Republic of Congo-DRC (former Zaire) in 1976 [6]. Since then, over 25 outbreaks have been reported in Africa, with most of them occurring in the Congo Basin [7]. The largest and deadliest outbreak ever registered occurred in 2014 and was caused by Zaire *ebolavirus*. It resulted in a very high case-fatality ratio of up to 90% [8]. Ebola virus has largely circulated in sub-Saharan Africa causing dreadful outbreaks of EVD [7]. Following the West African outbreak in 2014, it has become a global public health security threat [9].

Fruit bats (Pteropodidae family) have been identified as the only reservoir of Ebola virus [10] though there is a possibility that other animals could also harbour the pathogen. Spread of infection to humans (primary transmission) occurs by the spillover effect, following contact with blood, secretions, organs and bodily fluids of an infected reservoir or an infected non-human primate [8, 11–13]. Person-to-person transmission (secondary transmission) occurs in the community following contact with blood, secretions or other bodily fluids of infected individuals, EVD patients or individuals who have died of Ebola. Healthcare workers in close contact with an Ebola patient without using appropriate infection control measures and adequate barrier procedures have been infected while treating patients [14]. Ebola virus can survive in liquid or dry material for days [15] facilitating transmission by fomites.

Many factors increase the risk of acquiring and transmitting Ebola virus [16]. Social conditions such as human mobility, behavioural and cultural practices, bushmeat consumption, burial practices, preference for traditional medicines and cures, and fear and obstruction of health interventions have greatly enabled and enhanced human to human transmission [12, 17].

At the time of our study, there was no licensed treatment or vaccine for EVD. Patients were only given symptomatic treatment which when administered early could improve the chances of recovery [18]. Prevention and control measures of an outbreak of EVD are aimed at interrupting transmission. This is largely through avoidance of practices that predispose to infection [12]. For these measures to be successful there is a need for an understanding and avoidance of risky behaviours of a community. However, U.S. Food and Drug Administration (FDA), recently approved the rVSV-ZEBOV vaccine, a single dose vaccine that offers prevention against the *Zaire ebolavirus* species [19].

EVD outbreaks have been reported in some countries that border Cameroon [20]. Although the disease has never been reported, there is serologic evidence of Ebola virus in Cameroon [4, 21, 22] with highest rates of seropositivity among the pygmies (Baka people) and rain forest farmers (Bantu people) [21]. In a largescale survey of non-human primates across Central Africa, Leroy et al. [23] reported serologic evidence of exposure to Ebola infection in Chimpanzees in Cameroon. These findings confirm exposure to the Ebola virus and show that a less-virulent virus could be circulating in Cameroon, accounting for the absence of human cases and/or observed epizootics. The Southeastern equatorial rain forest of Cameroon harbours fruitbats which are reservoirs of Ebola virus, as well as animals susceptible to Ebola virus disease [24]. Being that this is part of the tropical rainforest of the Congo Basin; inhabitants of this area are at a high risk of exposure to the virus. In addition, studies have revealed a high exposure to non-human primates in Cameroon [25]. A larger investment is needed for containing rather than preventing an Ebola virus disease outbreak; prevention is therefore preferable to containment in areas at risk such as the rainforest of Cameroon. For prevention to be effective there is need for data to guide the design of health promotion interventions. It was against this background that we assessed the risk of exposure of the Baka community of Abong-Mbang Health District, South Eastern Cameroon to Ebola virus infection by investigating their knowledge of EVD and practices that could expose them to infection.

## Methods

### Study design and setting

This was a community based cross-sectional descriptive study carried out in Abong-Mbang Health District, Upper Nyong Division of East Cameroon. Abong-Mbang Health District is located in the south eastern rain forest of Cameroon which is part of the rainforest of Central Africa, where most Ebola virus disease outbreaks, except the 2014 West African outbreak originated [7, 26].

Abong-Mbang has an estimated population of about 28,904 inhabitants and covers an area of about 15.000 km^2^. It is made of 92 villages that are grouped into 8 health areas, Mindourou, Nkouak, Mbomba, Angossas, Ankoung, Atok, Abong-Mbang North and Abong-Mbang Southand has 25 public and private health facilities. Abong-Mbang has a wet equatorial climate (also known as a Guinea type climate). Its forest has abundant and diverse animal life with animals such as monkeys, some of the last populations of gorillas and chimpanzees [26]. Fruit and insectivorous bats and birds of various species are also common, as are various rodents.

### Study population

The study population comprised of the Baka community of Abong-Mbang Health District. This community is made up of 24 villages: Ampele, Andoa, Aviaton, Bitsoman, Cyrie, Djibot, Dympam, Diassa, Elandjoh, Kendjo, Madouaite, Mapela, Mayos, Mballam, Mbang, Mbiatoh, Mengang, Menzoh, Missoume, Moangong, Nombakele, Petit Paris, Plateau and Sombou; inhabited by two ethnic groups, Baka and Bantu. Out of the 24 villages in Abong-Mbang, our study involved 13 villages.

The Baka people (formerly called the Pygmies) are an ethnic group inhabiting the southeastern rain forests of Cameroon, northern Republic of the Congo, northern Gabon, and southwestern Central African Republic and Western Equatorial state of South Sudan. They are semi-sedentarised, spending part of the year in their roadside settlements and go for short- and long-term (up to several months) hunting and gathering expeditions deep into the forest. Most of them rely almost exclusively on traditional health care [27]. A few Bantu people reside in the study area, where they carryout mainly subsistence farming.

### Inclusion and exclusion criteria

Individuals aged ≥18 years who have resided for at least 5 years in the Baka community of Abong-Mbang Health District, and who granted consent to participate in the study were recruited. Those who denied consent/assent, were < 18 years old or had lived in the community for less than 5 years were excluded.

### Sampling and sample size determination

A multi-stage cluster sampling technique was used. Of the 24 villages of the Baka community 13 were randomly selected. From each of these villages, household heads or their representatives were recruited by systematic random sampling.

The minimum sample size was estimated using a single population proportion formula: *n*= $\frac{\mathrm{Z}2\ \mathrm{P}\left(1-\mathrm{P}\right)}{\mathrm{d}2.}$

Since there has been no similar study in Cameroon, the following assumptions were made: 95% (Z = 1.96) confidence level, 50% proportion and 5% margin error. Therefore *n=* $\frac{(1.96)(1.96)\mathrm{X}\ 0.5\left(1-0.5\right)}{(0.05)(0.05)}=499.41$. This was rounded up to 500 and 10 added to make up for non-responses giving a total sample size of 510 participants.

### Sample collection and analyses

Data was collected by trained research assistants using a pre-tested semi-structured questionnaire (Supplementary File 1) adapted from the risk factors involved in the Health Promotion Theory and then developed according to the objectives of the study. The questionnaire was divided into four sections: demographics, an assessment of participants’ knowledge of Ebola, their feeding habits and practices, and health seeking behavior as contributory factors to exposure to Ebola.

Data was entered into EPI Info 7, cleaned and analyzed using SPSS version 20.0. The relationship between the study outcome and the independent variable was analyzed using the Chi-square test. An adapted 8 item Morisky Scale was used to assess respondents’ level of knowledge of symptoms and transmission of Ebola. Participants who could list 4–8 correct manifestations or routes of transmission were considered to have good knowledge; those with 2–3 correct manifestations had fair knowledge and those who had one or none correct had poor knowledge.

An adapted Threat Capability Basic Risk Assessment Guide [28] was used to assess the level of risk involved in consuming bush meat. This guide had four different levels of assessment: very high, high, moderate and low. Participants who consumed all six animals listed (Fruit bats, chimpanzees, gorillas, bush pigs, monkeys, forest Antelopes, and Porcupines) were considered to be at a very high risk, 4–5 animals at high risk, 2–3 animals at moderate risk and 0–1 animal at low risk.

### Ethical considerations

Ethical approval was obtained from the Centre Regional Ethics Committee for Human Health Research (N^o^: CE031/CRERSHC) of the Ministry of Public Health, Cameroon. Administrative approval was obtained from the Regional Delegation of Public Health for the East Region. Verbal informed consent was obtained from every participant prior to collection of data. This is because most of our participants could not read or write. Participants 21 years and above granted consent to participate in the studythose < 21 years old (minors) granted assent while consent for them to participate in the study was obtained from their parent/guardian. Interviews were conducted in private. Questionnaires were assigned codes instead of writing the name of the participants. The original questionnaire which was in English was translated to French. Data collectors were French speaking and three of them also served as translators as they could speak the Baka language.

## Results

### Characteristics of study population

A total of 510 individuals participated in this study. Males (49.6%) and females (50.4%) were almost of the same proportion. Participants were from two ethnic groups: Baka (68%) and the Bantu (32%). The highest proportion were natives of the study area (96.5%), had primary level of education (62.7%), aged 18–25 years (32.2%), married (73.5%), hunters (31.4%) and had 1–5 children (72.5%) (Table 1).

**Table 1:** Baseline characteristics of study Population

Characteristic	Number (N^**o**^)	Percentage (%)
**Ethnic group**		
Baka	347	68**%**
Bantu	163	32**%**
**Sex**		
Male	253	49.6**%**
Female	257	50.4**%**
**Age**		
18–25	164	32.2**%**
26–36	147	28.8**%**
37–47	70	13.7**%**
48–58	63	12.4**%**
> 58	66	12.9**%**
**Level of Education**		
No Education	102	20.0**%**
Primary	320	62.7**%**
Secondary	80	15.7**%**
Tertiary	8	1.6**%**
**Marital Status**		
Single	102	20.0**%**
Married	375	73.5**%**
Widow	33	6.5**%**
**Native/Non-native of Baka village**		
Natives	492	96.5**%**
Non-natives	18	3.5**%**
**Number of children**		
(1–5) Children	301	72.5**%**
(6–10) Children	95	22.9**%**
≥ 11 Children	19	4.5**%**
**Occupation**		
Hunter	160	31.4**%**
Farmer	152	29.8**%**
Both (Farmer and Hunter)	99	19.4**%**
Traditional healer	19	3.7**%**
Others	80	15.7**%**

### Knowledge of Ebola and relationship with demographic characteristics of participants

**Fig. 1:**
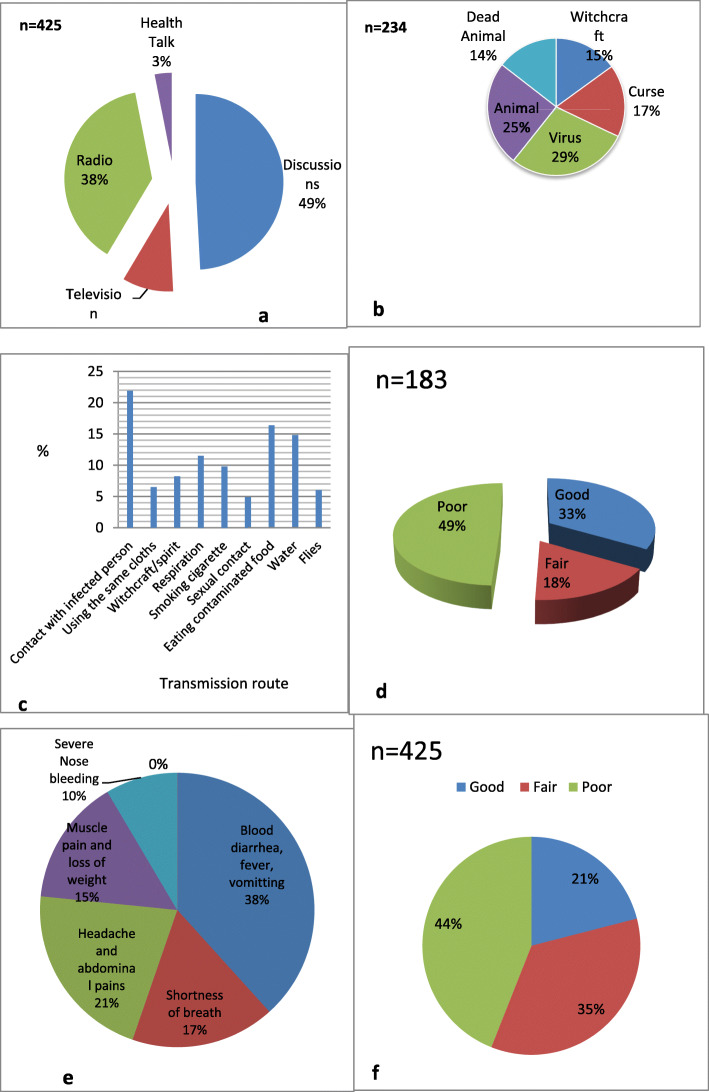
Participants’ knowledge of EVD (**a**) Sources of information of study participants (**b**) Opinion on causes of EVD; (**c**) Modes of Transmission(**d**) Manifestations encountered by participants (**e**) Level of knowledge of means of EVD Transmission (**f**) Level of knowledge of manifestations of Ebola

Four hundred and twenty-five (83.3%) participants had heard of Ebola. Their sources of information were: discussions among community members (49%), radio (38%), television (10%) and health talk (3%) (Fig. 1a). There was no significant difference in the level of awareness of participants on Ebola with respect to ethnic group (χ^2^ = 2.469 *P* = 0.116), gender (χ^2^ = 1.319 *P* = 0.251) and age (χ^2^ = 6.418 *P* = 0.170). Significant differences were observed with respect to level of education (χ^2^ = 408.00 *P* < 0.05) and occupation (χ^2^ = 483.474, *P <* 0.05) (Table 2).

**Table 2:** Relationship of demographic characteristics of study participants and their awareness on Ebola

Demographic Characteristics	Have you ever heard of Ebola 510(100%)		Knows what causes/gives Ebola? 425(83.3%)		Believe Ebola can be transmitted from one person to another 425(83.3%)		
Total	No N (^%^)	Yes N (^%^)	No N (^%^)	Yes N (^%^)	No N (^%^)	Yes N (^%^)	No Idea N (^%^)
	85 (16.7)	425 (83.3)	191 (44.9)	234 (55.1)	146 (34.4)	183 (43.1)	96 (22.6)
**Ethnic group**	***P =*** **0.116**		***P <*** **0.05**		***P <*** **0.05**		
Baka	64 (12.5)	283 (55.5)	125 (29.4)	158 (37.2)	72 (16.9)	142 (33.4)	69 (16.2)
Bantu	21 (4.1)	142 (27.8)	66 (15.5)	76 (17.9)	74 (17.1)	41 (9.7)	27 (6.4)
**Sex**	***P =*** **0.251**		***P =*** **0.199**		***P*** **= 0.087**		
Male	47 (9.2)	206 (40.4)	86 (20.2)	120 (28.2)	75 (17.7)	94 (22.1)	37 (8.7)
Female	38 (7.5)	219 (42.9)	105 (24.7)	114 (26.8)	71 (16.7)	89 (20.9)	59 (13.9)
**Age**	***P =*** **0.170**		***P*** **= 0.744**		***P*** **= 0.236**		
15–25	26 (5.1)	138 (27.1)	57 (13.4)	81 (19.1)	35 (8.2)	68 (16)	35 (8.2)
26–36	26 (5.1)	121 (23.7)	57 (13.4)	64 (15.1)	49 (11.5)	44 (10.4)	28 (6.9)
37–47	11 (2.2)	59 (11.6)	29(6.8)	30 (7.1)	21 (4.9)	25 (5.9)	13 (3.1)
48–58	16 (3.1)	47 (9.2)	23 (5.4)	24 (5.7)	20 (9.7)	17 (4)	10 (2.4)
> 58	6 (1.2)	60 (11.8)	25 (5.9)	35 (8.2)	21 (4.9)	29 (6.8)	10 (2.4)
**Education**	***P <*** **0.05**		***P <*** **0.05**		***P <*** **0.05**		
No Education	73 (14.3)	29 (5.7)	25 (5.9)	74 (17.4)	55 (12.9)	30 (7.1)	17 (4)
Primary	10 (2.0)	310 (60.8)	96 (22.9)	142 (33.4)	82 (19.3)	110 (25.9)	35 (8.2)
Secondary	2 (0.4)	78 (15.3)	70 (16.5)	10 (2.4)	9 (2.1)	35 (8.2)	44 (10.4)
Tertiary	0 (0)	8 (1.6)	0 (0)	8 (1.9)	0 (0)	8 (1.9)	0 (0)
**Occupation**	***P <*** **0.05**		***P <*** **0.05**		***P <*** **0.05**		
Hunter	33 (6.5)	129 (25.3)	61 (14.4)	50 (11.8)	65 (15.3)	45 (10.6)	20 (4.7)
Farmer	19 (3.7)	133 (26.1)	43 (10.1)	74 (17.4)	50 (11.8)	43 (10.1)	30 (7.1)
Both (hunter and farmer)	20 (3.9)	79 (15.5)	66 (15.5)	32 (7.5)	17 (4)	40 (9.4)	32 (7.5)
Traditional healer	3 (0.6)	16 (3.1)	1 (0.2)	18 (4.2)	5 (1.2)	14 (3.3)	0 (0)
Others	10 (2.0)	68 (13.3)	20 (4.7)	60 (14.1)	9 (2.1)	41 (9.7)	14 (3.3)

Out of the 425 respondents who had heard of Ebola, 234 (55.1%) reported that they knew its cause. The highest proportion (29%) reported a virus as the causative agent. Other causes reported were animal (25%), dead animal (14%), witchcraft (15%) and curse (17%) (Fig. 1b). There were significant differences in level of knowledge of causes of Ebola with respect to ethnic group (χ^2^ = 44.270 *P* < 0.05), level of education (χ^2^ = 170.848 *P <* 0.05) and occupation (χ^2^ = 271.534, *P <* 0.05) (Table 2).

Only 183 (43.1%) of the 425 participants who had heard of Ebola believed that it could be transmitted from person-to-person. One hundred and forty-six (34.4%) denied the possibility of human-to-human transmission while the remaining 96 (22.6%) had no idea of transmission. Contact with an infected person (21.8%) was the mode of transmission reported by most participants (Fig. 1c). Other modes of transmission reported were: eating contaminated food (16.4%), water (14.8%), respiration (11.5%), smoking cigarette (9.8%), witchcraft/spirit (8.2%) and flies (6%) (Fig. 1c). Most participants (49%) had poor knowledge of transmission of Ebola virus disease (Fig. 1d). Ethnic group (χ^2^ = 30.751 *P <* 0.05), level of education (χ^2^ = 381.370, *P <* 0.05) and occupation (χ^2^ = 644.521, *P <* 0.05) showed significant differences with respect to knowledge of modes of transmission of Ebola (Table 2).

With regards to manifestations of EVD, bloody diarrhea, fever and vomiting were the symptoms most reported (38.3%) (Fig. 1e). Participants who reported these symptoms were mainly traditional healers and they indicated that some patients with such manifestations had come to their shrine for consultation. Other participants saw similar symptoms in the health center. One man reported he came across a patient, vomiting blood with hiccups in a hospital in Bertoua and it was rumoured the patient was suffering from Ebola hemorrhagic fever. Some respondents reported they lost a relative who had suffered from bloody diarrhea and high fever for 1–2 weeks. Among respondents who had heard about Ebola, 11% (47) had come across a person suffering from at least one of the manifestations listed and 5.3% indicated that the manifestations were common in the community. Based on the Morisky Scale the majority of participants (44%) had poor knowledge of the symptoms of Ebola (Fig. 1f).

### Feeding behaviour

**Fig. 2:**
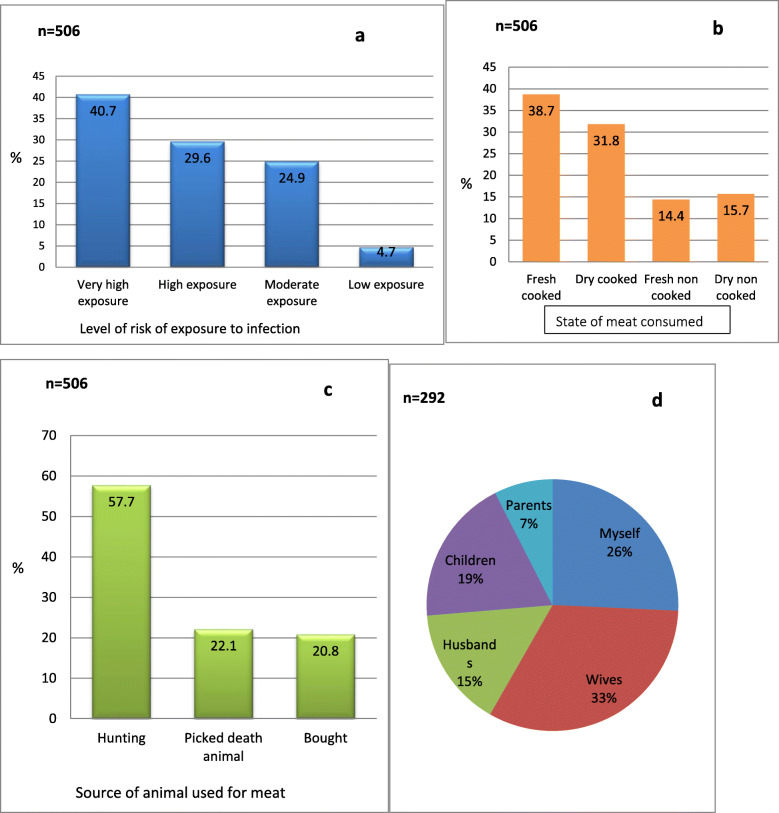
Feeding behaviour of participants: (**a**) Risk of exposure of participants to Ebola virus infection (**b**) State of meat consumed (**c**) Source of meat (**d**) Person preparing the meat

Consumption of bushmeat was a common practice among respondents as 506 (99.2%) consumed bush meat. Animals mostly consumed were fruit bats, chimpanzees, gorillas, bush pig, monkeys, forest antelope and porcupines. Based on the level of risk of exposure to infection, 40.7, 29.6, 24.9 and 4.7% of participants respectively were considered to be at a very high risk, high risk, moderate risk and low risk of exposure (Fig. 2a). Participants consumed cooked fresh meat (38.7%) or cooked dry meat (31.8%), however, 14.4% consumed fresh uncooked meat (Fig.2b). The meat was mostly hunted (57.7%). Some respondents (22.1%) consumed dead animals recovered from the forest (Fig. 2c). As the underlying risk of exposure to infection with Ebola virus is on the person who prepares the meat due to direct contact with the blood/body fluid of the animal, based on our investigation meat preparation was done mainly women (wives of the male respondents) (33%) (Fig. 2d).

### Health-seeking behaviour

**Fig. 3:**
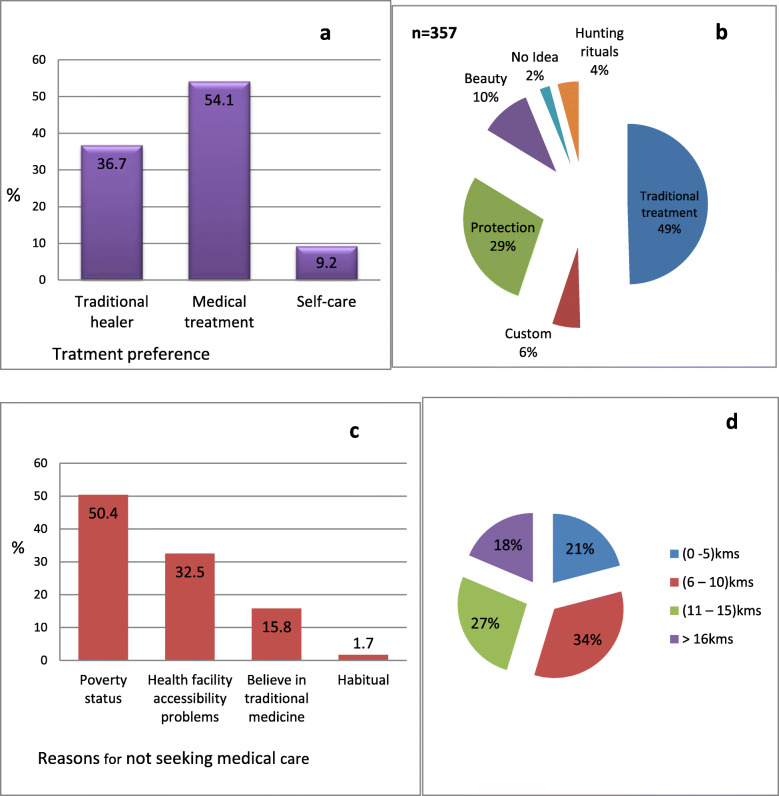
Health seeking behaviour of participants (**a**) Treatment preferences of study participants (**b**) Reasons for scarification (**c**) Reasons for not seeking medical care in the health facility (**d**) Distance to health facility from place of residence

The health seeking behavior of participants was investigated to highlight its role on the spread of the Ebola virus (Fig. 3). Factors influencing health seeking behavior such as socio-cultural and service-related factors were investigated. Socio-cultural factors included beliefs about illness etiologies and trajectories, treatment strategies (scarifications), treatment preferences (medical treatment, traditional healer and self-care), and characteristics of the individuals engaged in health seeking (level of education, poverty status). The majority of respondents (54.1%) sought health care in a medical facility when sick. However, 36.7 and 9.2% consulted traditional healers and provided self-care respectively (Fig. 3a).

Three hundred and fifty-seven (70%) respondents had been scarified. Scarifications were performed mainly for traditional treatment (49%), spiritual protection (29%) and for aesthetic reasons (10%) (Fig. 3b). Of the 234 respondents who did not seek medical care when sick, about half of them (50.4%) indicated financial constraints as a deterrent factor (Fig. 3c). Other major reasons advanced were inaccessibility of health care facility (32.5%) and belief in traditional medicine (15.8%). However, the majority of respondents (91.6%) indicated they had no health facility in their village of residence. Thirty-four percent (34%) of participants travelled a distance of 6–10 km, to get to a health facility while only few (21%) covered ≤5 km. The rest covered distances ≥11 km to get to a health facility (Fig. 3d).

The attitude of health personnel towards respondents and the functionality of the health facility were also discouraging factors to seeking medical treatment. Participants reported they were at times ignored by health staff because of their social and poverty status. Thus, the intrusive nature of health staff towards these individuals was a potential contribution to them not seeking medical treatment. In one of the villages, the health center had not been functional for the 3–4 years prior to this study due to lack of equipment and staff.

## Discussion

EVD outbreaks constitute a major global public health concern [9, 29]. Since the first outbreak of Ebola reported in 1976, globally, there have been over 36 documented outbreaks (19 major outbreaks and 17 minor) [30]. The largest outbreak lasted from 2014 to 2016 in West Africa and resulted in 28,646 cases and 11,323 deaths [31]. At the time of this study, there was an ongoing outbreak of Ebola in DRC with increasing number of cases [32] and it extended to Uganda [33]. To minimize the chances of an outbreak in areas at risk such as the Baka community, knowledge of factors that could predispose inhabitants to infection is necessary. WHO aims to prevent Ebola outbreaks by maintaining surveillance for EVD and supporting at-risk countries to develop preparedness plan. Prevention can only be successful following an understanding of potential risk factors as this will be useful to develop intervention measures targeting communities at risk. It was against this background that this study was carried out in the Baka community.

Most participants (83.3%) had heard of Ebola virus disease. The level of awareness in our study was lower than 96% reported in Guinnea [34] and 88% in Sokoto, Nigeria [35]. These studies and ours were conducted during the West African EVD outbreak explaining the high level of awareness. Compared to other countries where information on the disease was obtained mainly through mass media [36, 37], in our study, participants learned of the disease mainly from discussions in the community. Health talk was reported by a very small proportion (3%) of participants. This is disturbing as informal discussions in a community with such a low level of education as observed in the Baka community could have far reaching consequences as wrong information could be circulated and in the event of an outbreak of EVD, such information may contribute to more exposures to infection. Proper education of inhabitants of the Baka community through health talk is therefore very important. Radio signals are widely captured in the study area, explaining why radio was another main source of information. Those who had heard about Ebola through television were individuals who had visited the urban areas as television signals were poor in the villages studied.

With regards to symptoms, bloody diarrhea, fever and vomiting were reported by most of our participants. Evaluating the level of knowledge of symptoms, only few participants had good knowledge. This indicates the need for an intensive sensitization campaign in the study area, particularly as EVD has often been reported in some countries in the Congo Basin. Proper education of inhabitants in an area at risk such as the Baka community through health talks is very important as these are given by individuals who are well informed about the disease. Among participants who had heard of Ebola slightly more than half reported they knew the cause of the disease of which less than one-third stated a virus as the cause. This is not surprising because with the low level of education of participants, they could easily remember the factors that facilitate transmission such as animal or dead animal instead of the virus as the cause of EVD. Based on our study, participants’ knowledge of the cause, transmission and manifestation of EVD was poor. However, the Baka people had significantly higher knowledge of these than the Bantus. Being hunter-gathers with a high exposure to animals compared to the Bantu people who are mainly subsistence farmers, could have made it easier for the Baka people to recall information on the cause of EVD and the role of animals in transmission as this pertained to their main occupation.

There were misconceptions on the cause and transmission of the disease, as some participants attributed it to a curse or witchcraft and others rejected the possibility of human-to-human transmission of EVD. Some participants attributed transmission to respiration, eating contaminated food, smoking cigarette, water and flies. Similar misconceptions have been reported in areas with an Ebola outbreak [34–38] and underscore the need for intensive health promotion efforts in this community particularly as only 33% had good knowledge of transmission. Upon completion of our study, community sensitization was done in study area in collaboration with FAIRMED (formerly Leprosy Relief Emmaus Switzerland), under a self-help-oriented project implemented by FAIRMED Cameroon, with the aim of improving the health and empowering members of this community. Also, the findings of this research were communicated to health practitioners, policymakers, and the public through the Abong-Mbang Health District monthly coordination and health dialogue structure meetings. If we have funding, we will design and carry out a follow-up study to determine whether there has been an increase in knowledge and change in behaviour so as to further correct misconceptions and risky practices observed.

Studies have shown ethnic background to be an important risk factor influencing exposure to Ebola virus in many communities [21]. Cultural beliefs and behaviours have accounted for the persistence of outbreaks as they counter prevention and control measures [16, 37–41]. We investigated the feeding behavior and health seeking behavior of the inhabitants of the Baka community to understand how they could contribute to exposure to Ebola virus infection. Bush meat hunting and consumption was a common practice among inhabitants with some consuming uncooked fresh meat. Hunting is not only for domestic use but is also an income generating activity in the study area. As previous reports have shown evidence of Ebola virus infection in some of the animals consumed [22], these are practices that could expose them to EVD if the strain in circulation is virulent. The majority of participants (40.7%) were considered to be at a very high risk of exposure to infection. Bush meat handling, preparation and consumption has been recognized as an important contributor to the spillover effect in areas that have witnessed an outbreak of Ebola [12, 13]. Despite this high exposure, no case of Ebola virus disease has been documented in Cameroon though there are reports on evidence of infection in humans [20, 42, 43]. These studies reported highest rates of infection among pygmies, young adults, and rainforest farmers [20]. This shows that a less virulent strain might have circulated in study area and could not cause any clinical disease.

The health seeking behaviour was investigated to highlight its role in the secondary transmission of infection. Although more than half of the participants reported they seek medical treatment when ill, a significant proportion relied on traditional medicine and self-care. Studies in areas that have witnessed an outbreak of Ebola [34, 35] have reported higher proportions of participants seeking medical care if they fall ill. This is because they must have received health promotion messages which emphasized the importance of medical care. The Baka are renowned for traditional healing [44]. In our study, almost two-thirds of participants had undergone scarification which was performed for treatment purposes. Other major reasons for scarification were spiritual protection, customary practice and beauty enhancement. In case of an outbreak, practices such as scarification could play a major role in secondary transmission of EVD. This is because with their low level of education, traditional healers may have limited knowledge of infection prevention and may instead expose their patients to infection. The semi-sedentary lifestyle of the Baka people influenced their ability to seek medical treatment in a health facility particularly if it is at a distant location. Poverty, health care accessibility and functionality were the major contributory factors. Up to 91.2% of study participants indicated they did not have a health facility in their village of residence. In one of the villages, the Health Centre is present but had not been functional for some years due to lack of equipment and staff. Some participants complained of the attitude of some medical staff who at times ignored them because of their social and economic status. This greatly discouraged them from subsequent visits.

Our study did not complement the quantitative data with sufficient qualitative data to capture more information on knowledge, practices and predisposing factors to Ebola virus infection and spread. In addition, the design of the questionnaire limited the amount of data that was collected on knowledge and behavioural practices that could influence exposure. These constituted limitations to our study.

## Conclusion

Although the majority of the Baka community was aware of EVD, their knowledge on its cause and transmission was poor. Most participants had misconceptions about EVD and were engaged in practices that could expose them to infection with Ebola virus. Based on our findings there is need for rigorous sensitization to educate people about Ebola virus disease and clarify the misconceptions observed among participants. Surveillance of communities in study area for EVD is recommended particularly as outbreaks of EVD have often been reported in the Congo Basin. There is an urgent need for more functional medical facilities in study area for prompt disease diagnosis and management.

## Supplementary Information

**Additional file 1.** Questionnaire. Questionnaire administered to study participants.

## Data Availability

The datasets used and/or analysed during the current study are available from the corresponding author on reasonable request

## Abbreviations

CDC: Centers for Disease Control and Prevention
DRC: Democratic Republic of Congo
EVD: Ebola virus disease
EV: Ebola virus
WHO: World Health Organization
