# Supplementary figures and images for: Assessing the determinants of Ebola virus disease transmission in Baka Community of the Tropical Rainforest of Cameroon
Source: BMC Infect Dis. 2021 Apr 7;21:324. doi: 10.1186/s12879-021-06011-z (PMC8028822; doi:10.1186/s12879-021-06011-z)

# Questionnaire (English version)


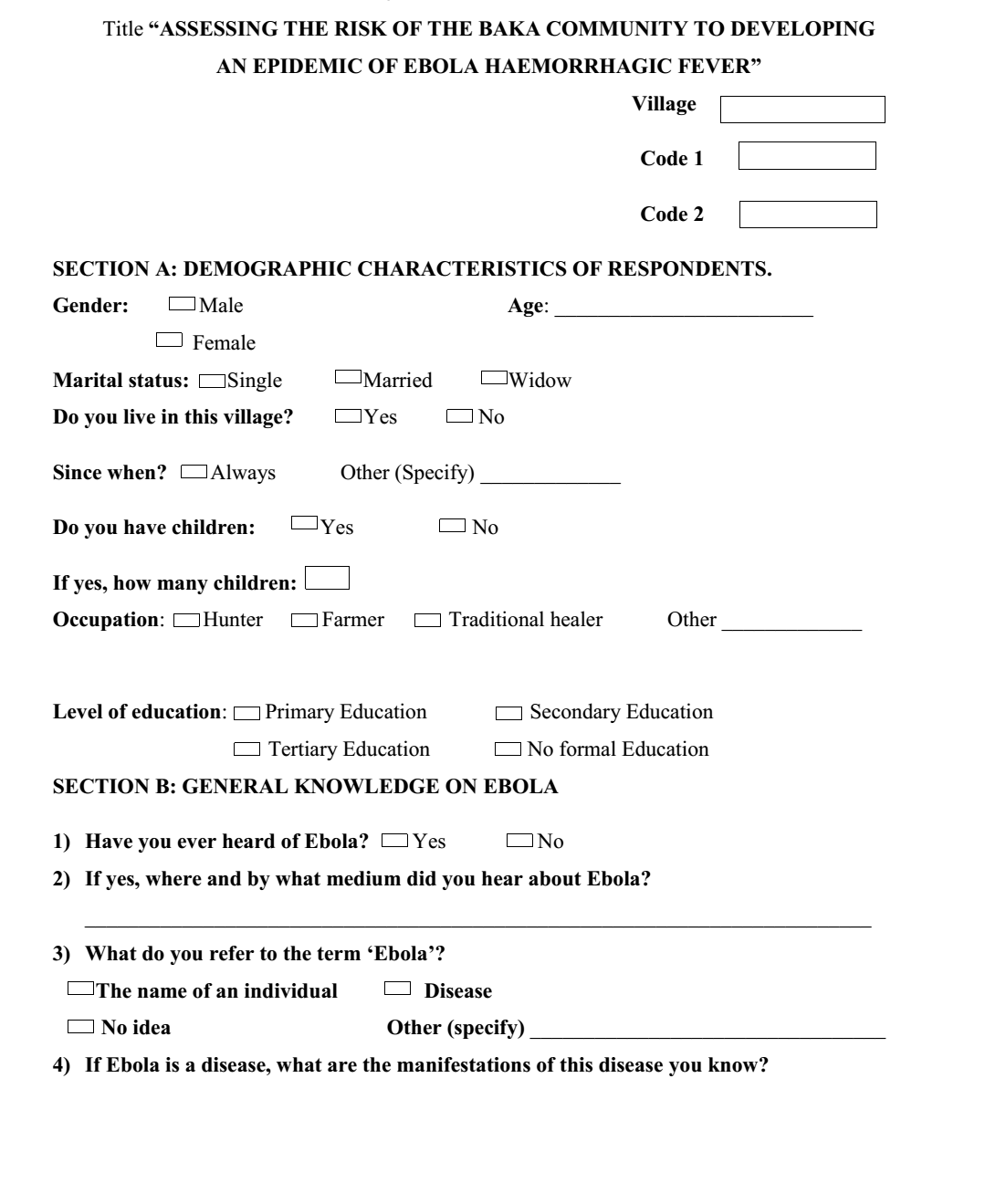


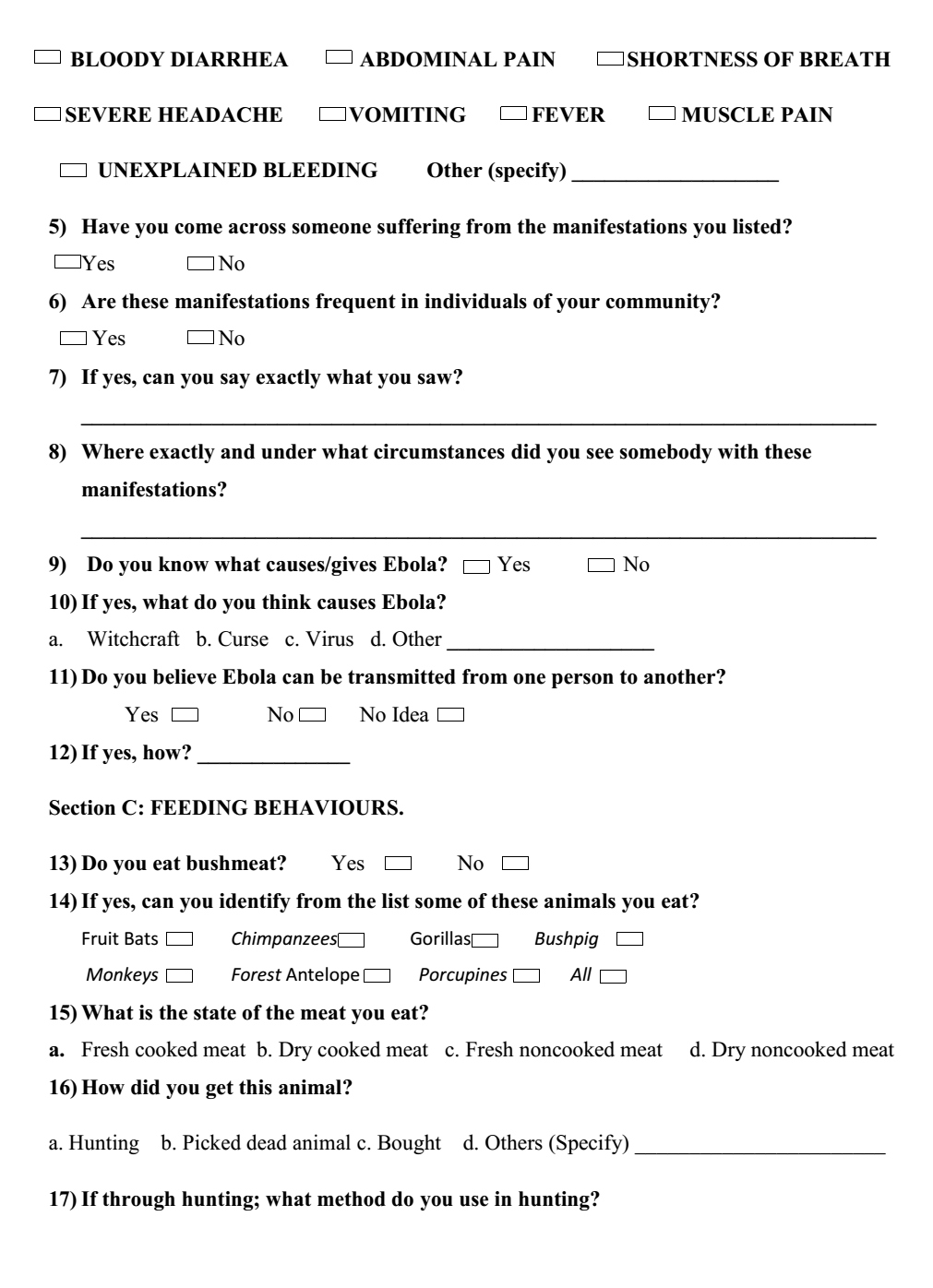


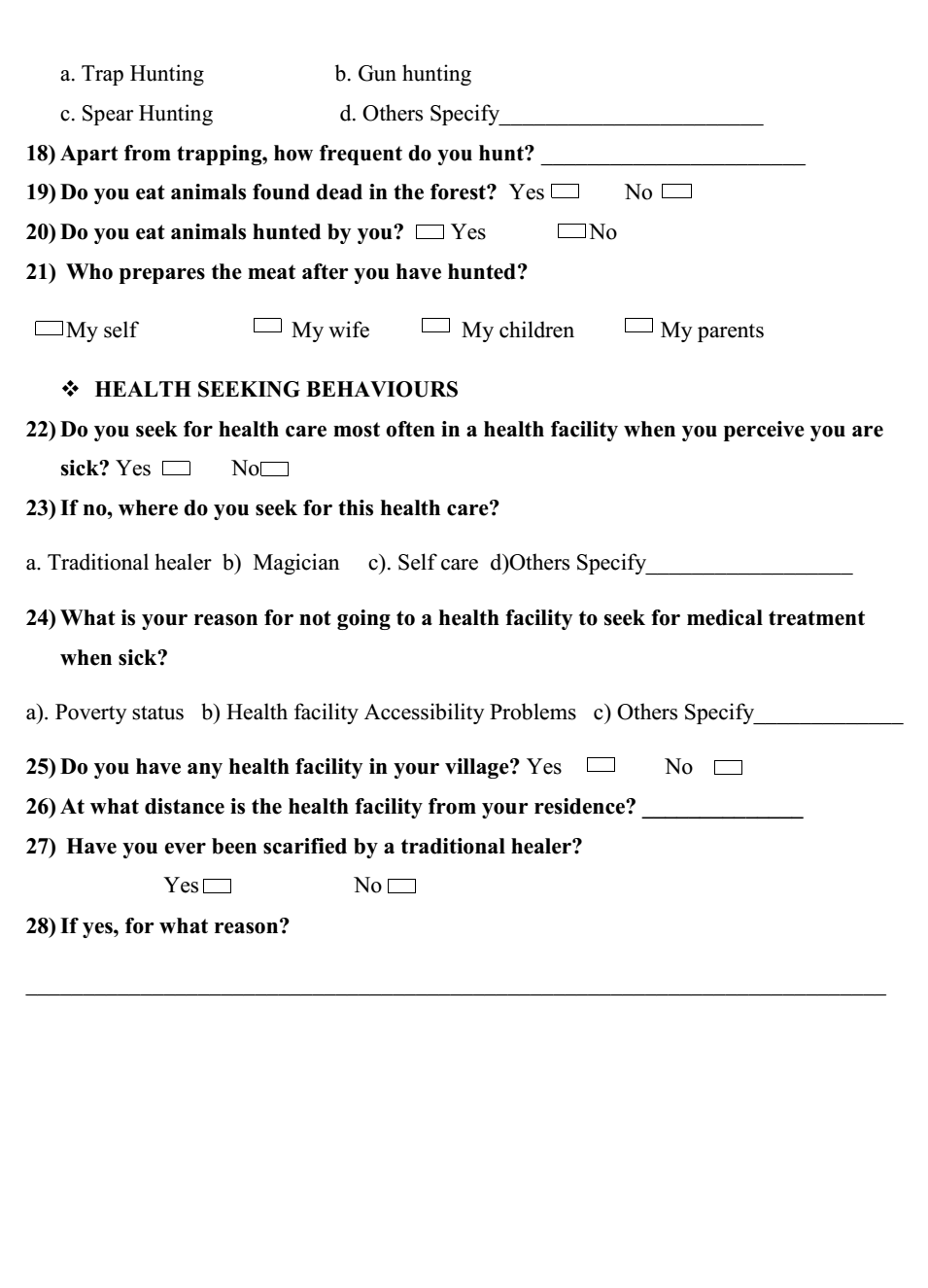

Supplement: Supplementary file 1 — Additional file 1. Questionnaire. Questionnaire administered to study participants. [file 12879_2021_6011_MOESM1_ESM.docx]
